# Effective Properties of Homogenised Nonlinear Viscoelastic Composites

**DOI:** 10.3390/ma16113974

**Published:** 2023-05-25

**Authors:** Alejandro Roque-Piedra, Reinaldo Rodríguez-Ramos, Raimondo Penta, Ariel Ramírez-Torres

**Affiliations:** 1 School of Mathematics & Statistics, University of Glasgow, University Place, Glasgow G12 8QQ, UK; 2 Facultad de Matemática y Computación, Universidad de La Habana, San Lázaro y L, Vedado, La Habana 10400, Cuba; 3 Programa de Pós-graduação Acadêmico (Stricto Sensu) em Modelagem Computacional em Ciência e Tecnologia (PPG-MCCT) da EEIMVR, Escola de Engenharia Industrial Metalúrgica de Volta Redonda da UFF, Universidade Federal Fluminense, Av. dos Trabalhadores 420, Vila Sta. Cecília, Volta Redonda 27255-125, RJ, Brazil

**Keywords:** nonlinear viscoelasticity, asymptotic homogenisation, anti-plane problem, effective coefficients

## Abstract

We develop a general approach for the computation of the effective properties of nonlinear viscoelastic composites. For this purpose, we employ the asymptotic homogenisation technique to decouple the equilibrium equation into a set of local problems. The theoretical framework is then specialised to the case of a strain energy density of the Saint-Venant type, with the second Piola–Kirchhoff stress tensor also featuring a memory contribution. Within this setting, we frame our mathematical model in the case of infinitesimal displacements and employ the correspondence principle which results from the use of the Laplace transform. In doing this, we obtain the classical cell problems in asymptotic homogenisation theory for linear viscoelastic composites and look for analytical solutions of the associated anti-plane cell problems for fibre-reinforced composites. Finally, we compute the effective coefficients by specifying different types of constitutive laws for the memory terms and compare our results with available data in the scientific literature.

## 1. Introduction

Viscoelasticity is a unique property exhibited by certain materials, which encompasses a combination of viscous and elastic behaviours during deformation. Unlike purely viscous materials, viscoelastic bodies exhibit time-dependent behaviour when subjected to constant stress, deforming at a consistent rate.

The study of the mechanical properties of viscoelastic composites has been of great interest due to their unique characteristics. There is a large amount of scientific literature addressing the homogenised properties of viscoelastic composites using different homogenisation approaches [1,2,3,4,5], including those within a variational setting [6,7,8,9]. In the context of the asymptotic homogenisation technique, investigations have mainly focused on linear viscoelastic materials since one can exploit the correspondence principle introduced by the use of the Laplace transform. For instance, in [10], the Authors obtain the effective properties of linear viscoelastic composites and focus on the role of memory effects. Moreover, in [11] the two-scale asymptotic homogenisation technique together with FEM simulations are used to study the effective properties of non-ageing linear viscoelastic properties with different cell geometrical arrangements. Besides, in [12], a three-scale asymptotic homogenisation is used to model hierarchical viscoelastic materials. In [13], the Authors show the benefit of computing the viscoelastic and thermoviscoelastic properties of heterogeneous materials by means of an asymptotic homogenisation approach which treats the temperature as a parameter. On the other hand, in [14], the influence of fibre size and fibre-matrix interface adhesion on the effective mechanical properties of fibre-reinforced composites is investigated using a perturbation method of the boundary shape. Within the linear viscoelastic framework, in [15] the methodology described in [16,17] is extended to find closed-form expressions of the effective elastic coefficients of unidirectional fibre-reinforced materials to the case of non-ageing, viscoelastic composites.

Nonlinear viscoelastic materials have emerged as invaluable tools in various real-world applications, showcasing their remarkable utility. A prime example lies within the realm of 3D printing to replicate the intricate mechanical properties of biological tissues [18]. By harnessing their unique characteristics, nonlinear viscoelastic composites also enable researchers to mimic the behaviour of fibrous tissues [19] and aortic tissue [20]. Moreover, nonlinear viscoelastic composites, such as the bioactive glass/polysulfone combination, have been extensively studied for their potential in developing implants that establish strong bonds with biological tissues [21]. Although ongoing research and innovation have made significant advances, several challenges persist in the modelling of nonlinear composites. These include the conception of mathematical models capable of connecting the mechanical interactions both reciprocally and through the different scales at which each interaction occurs and optimising the design of nonlinear composites to achieve desired mechanical properties. In the latter scenario, it is crucial to have a methodology that can accurately describe the geometric aspects of the microstructure, and the asymptotic homogenisation technique offers a fundamental solution for this purpose.

We remark that there are just a few instances where the asymptotic homogenisation method has been used in a nonlinear setting (see, e.g., [22,23,24]). For instance, in [22], the Author proposed a procedure to investigate periodic hyperelastic composites at finite strains. Furthermore, in [23] the focus is on the description of flow, elastic deformation and transport in poroelastic media characterised by growth effects at the pore scale. Finally, In [24], the Authors explore the impact of the internal evolution in heterogeneous media, specifically that characterized by a stress-driven phenomenological flow rule for inelastic distortions, on the macroscopic behaviour of composites. The study investigates the interplay between the inelastic deformations and the overall macroscopic response of the composite. Although these previous works provide an initial foundation for studying nonlinear composites within the framework of asymptotic homogenization, they do not specifically address the incorporation of nonlinear viscoelastic constitutive laws.

Based on the above considerations, the most relevant objective of the present work is to provide a generalised framework based on the asymptotic homogenisation approach [22,24,25,26,27] to characterise the overall behaviour of nonlinear viscoelastic composites. This is, to the best of our knowledge, the main novelty of this study with respect to others in the scientific literature. Our purpose is also to start setting a modelling infrastructure to analyse the effective properties of synthetic composites and biological tissues.

The manuscript is organised as follows. In Section 2, we introduce the main notions corresponding to the kinematics of the heterogeneous material, as well as the notions of scales separation, local periodicity and macroscopic uniformity. In Section 3, we describe the main problem and its multiscale formulation which is framed in the context of nonlinear viscoelasticity. Furthermore, in Section 4, we present the asymptotic homogenisation scheme for the problem at hand and formulate the set of cell problems. Particularly, in Section 5, we specialise the theory for the case of the Saint-Venant strain energy density and make use of the correspondence principle by working in the limit of infinitesimal displacements (see Section 6). This hypothesis permits to obtain the classical cell and homogenised problems in linear viscoelasticity as a special case. In Section 7, we continue our analysis by considering the case of uniaxially fibre-reinforced composites and, taking inspiration from [15], we write short formulae for the effective coefficients associated with the anti-plane problem. Finally, in Section 8, we consider different constitutive models for the memory functions of the viscoelastic constituents and compare the results with data available in the scientific literature.

## 2. Theoretical Background

### 2.1. Kinematics

Let B be a body composed by two different constituents $B_{1}$ and $B_{2}$ such that Γ is the interface between $B_{1}$ and $B_{2}$, and ${\bar{B}}_{1}\cup {\bar{B}}_{2}=B$ and ${\bar{B}}_{1}\cap B_{2}=B_{1}\cap {\bar{B}}_{2}=\emptyset$, where the upper bar denotes the closure of the set. If $\chi :B\times [t_{0},t_{f}[\to S$ denotes the motion, where S is the three-dimensional Euclidean space and, $t_{0}$ and $t_{f}$ corresponds to the initial and final times, respectively; the current configuration of B at time *t* is described by $B_{t}:=\chi \left(B,t\right)$. Then, by taking the reference configurations of $B_{1}$ and $B_{2}$ to be $B_{R1}$ and $B_{R2}$, respectively, we set the current configurations of $B_{1}$ and $B_{2}$ to be $B_{1t}:=\chi_{1}\left(B_{R1},t\right)$ and $B_{2t}:=\chi_{2}\left(B_{R2},t\right)$ where $\chi_{1}:B_{R1}\times [t_{0},t_{f}[\to S$ and $\chi_{2}:B_{R2}\times [t_{0},t_{f}[\to S$ represent the restrictions of the motion to $B_{R1}$ and $B_{R2}$. In particular, we denote by $\Gamma_{R}$ the interface between $B_{R1}$ and $B_{R2}$, and consider that ${\bar{B}}_{R1}\cup {\bar{B}}_{R2}=B_{R}$ and ${\bar{B}}_{R1}\cap B_{R2}=B_{R1}\cap {\bar{B}}_{R2}=\emptyset$, where $B_{R}$ is the reference configuration of B. Then, the interface between $B_{1t}$ and $B_{2t}$ is $\Gamma_{t}:=\chi \left(\Gamma_{R},t\right)$ and is such that ${\bar{B}}_{1t}\cup {\bar{B}}_{2t}=B_{t}$ and ${\bar{B}}_{1t}\cap B_{2t}=B_{1t}\cap {\bar{B}}_{2t}=\emptyset$. For our purposes, we consider $B_{R2}$ to be characterised by the disjoint union ${⊔}_{k=1}^{N}B_{R2}^{k}$.

We specify by $X_{a}$ (a=1,2,3) the coordinates of a material point in the reference configuration $B_{R}$, and each spatial point with coordinates $x_{a}$ (a=1,2,3) is given by $x_{a}=\chi_{a}\left(X,t\right)$. We notice that we are tacitly assuming that the coordinates *x* and *X* are referred to the same orthogonal Cartesian system. In this setting, the deformation gradient tensor, F(X,t), can be defined as

(1) $$\begin{array}{c} F(X,t)=I+\mathrm{Grad}\;u(X,t), \end{array}$$

where I denotes the second-order identity tensor, u is the displacement vector and Gradu is the gradient of u with components ${\left[\mathrm{Grad}\;u\right]}_{ab}=\partial u_{a}/\partial X_{b}$.

### 2.2. Scales Separation

We denote the length scales characterising the composite medium and its internal structure by $L_{c}$ and *ℓ*, respectively, and introduce the smallness parameter, ε, as

(2) $$\begin{array}{c} 0<\varepsilon :=\frac{\ell }{L_{c}}\ll 1. \end{array}$$

Adopting the considerations in [28], if Φ represents a scalar field, or a component of a vector or tensor field, we can write Φ in its two-scale version as

(3) $$\begin{array}{c} \Phi \left(X,t\right)=\Phi^{\varepsilon }\left(\bar{X},\bar{Y},t\right), \end{array}$$

where $\bar{X}=X/L_{c}$ and $\bar{Y}=X/\ell =\bar{X}/\varepsilon$ are dimensionless variables usually referred to as the slow or macroscale variable and the fast or microscale variable, respectively. Consequently, by means of the chain rule, the partial derivative of Φ with respect to $X_{a}$ can be expressed as

(4) $$\begin{array}{c} \frac{\partial \Phi (X,t)}{\partial X_{a}}=\frac{1}{L_{c}}[\frac{\partial \Phi^{\varepsilon }\left(\bar{X},\bar{Y},t\right)}{\partial {\bar{X}}_{a}}+\frac{1}{\varepsilon }\frac{\partial \Phi^{\varepsilon }\left(\bar{X},\bar{Y},t\right)}{\partial {\bar{Y}}_{a}}]. \end{array}$$

In the following, for the sake of simplicity in our notation, we will simply write *X* and *Y* when referring to $\bar{X}$ and $\bar{Y}$, respectively.

### 2.3. Macroscopic Uniformity and Periodicity

**Figure 1:**
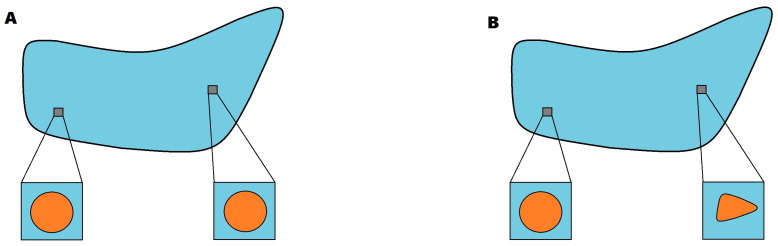
Sketch of the difference between the assumption of macroscopic uniformity (**A**) and non-macroscopic uniformity (**B**).

The scales separation induced by the macroscopic variable *X* and the microscopic variable *Y* permits selecting at the lower scale a unit cell Y, such that $Y={\bar{Y}}_{1}\cup {\bar{Y}}_{2}$, where, in the context of this work, we consider that $Y_{1}$ surrounds the inclusion $Y_{2}$ with $\Gamma_{Y}$ being the interface between $Y_{1}$ and $Y_{2}$. We notice that, in particular, $\partial Y_{1}=\partial Y\cup \Gamma_{Y}$ and $\partial Y_{2}=\Gamma_{Y}$, where ∂Y is the external boundary of the cell. In this work, we adopt the assumption of macroscopic uniformity (see, for instance [29,30,31] for further discussions). That is, we consider that the topological properties of the microscopic cell are independent of the macroscopic variable (Figure 1A).

There are two main consequences of the consideration of macroscopic uniformity. The first one is that we can select the unit cell independently of the position at the macroscopic domain and, thus, it is representative of the whole microstructure. The second consequence is that it allows to interchange the operator of differentiation with respect to the macroscopic variable with the integration over the repeating unit cell, namely,

(5) $$\begin{array}{c} \frac{\partial }{\partial X_{a}}(\int_{Y}\Phi^{\varepsilon }\left(X,Y,t\right)dV\left(Y\right))=\int_{Y}\frac{\partial \Phi^{\varepsilon }\left(X,Y,t\right)}{\partial X_{a}}dV\left(Y\right). \end{array}$$

While the above considerations are of great help when dealing with calculations, the assumption of macroscopic uniformity can be relaxed by paying the price of elevated computational cost (see, for example, [32,33]).

Finally, we will also consider that the fields of interest are locally periodic. Specifically, following [34], we introduce a family of vectors $r\left(\alpha_{1},\alpha_{2},\alpha_{3}\right)=\alpha_{i}e_{i}$, with $\alpha_{i}\in Z$ and ${\left\{e_{i}\right\}}_{i=1}^{3}$ being an orthonormal basis, such that $\Phi^{\varepsilon }\left(X,Y,t\right)=\Phi^{\varepsilon }\left(X,Y+r\left(\alpha_{1},\alpha_{2},\alpha_{3}\right),t\right)$.

## 3. Multiscale Formulation of the Problem

We set our problem within a purely mechanical framework by considering the equilibrium equation in the absence of body forces for each constituent, which, in its material form, can be written as

(6) $$\begin{array}{c} \mathrm{Div}\;P_{\eta }\left(X,t\right)=0, \end{array}$$

where $\left(X,t\right)\in B_{R\eta }\times [t_{0},t_{f}[$ with $0\leq t_{0}<t_{f}$, and $P_{\eta }$ denotes the first Piola–Kirchhoff stress tensor for each constituent, defined by η=1,2. Furthermore, Equation (6) is complemented with conditions of ideal contact at the interface Γ between $B_{R1}$ and $B_{R2}$, namely,

(7a) $$\begin{array}{cc} \; & u_{1}\left(X^{-},t\right)=u_{2}\left(X^{+},t\right), \end{array}$$

(7b) $$\begin{array}{cc} \; & P_{1}\left(X^{-},t\right)N\left(X\right)=P_{2}\left(X^{+},t\right)N\left(X\right), \end{array}$$

where $u_{\eta }$ represents the displacement vector and N is the normal vector pointing from $B_{R2}$ to $B_{R1}$. In Equations (7a) and (7b) the notations $\Phi_{1}\left(X^{-},t\right)$ and $\Phi_{2}\left(X^{+},t\right)$ are defined as

$$\begin{array}{c} \Phi_{1}\left(X^{-},t\right):=\lim_{X\to X^{-}}\Phi_{1}\left(X,t\right),\;\mathrm{and}\;\Phi_{2}\left(X^{+},t\right):=\lim_{X\to X^{+}}\Phi_{2}\left(X,t\right), \end{array}$$

where $X^{\pm }\in \Gamma_{R}^{\pm }$ with $\Gamma_{R}^{-}$ being the part of the interface $\Gamma_{R}$ in contact with $B_{R1}$ and $\Gamma_{R}^{+}$ the part of $\Gamma_{R}$ in contact with $B_{R2}$.

Recalling the scale separation introduced in Section 2.2 and the assumption of macroscopic uniformity, the problem specified by Equations (6), (7a) and (7b) takes the form

(8a) $$\begin{array}{cc} \; & L_{c}^{-1}\left[{\mathrm{Div}}_{X}P_{\eta }^{\varepsilon }\left(X,Y,t\right)+\varepsilon^{-1}{\mathrm{Div}}_{Y}P_{\eta }^{\varepsilon }\left(X,Y,t\right)\right]=0, \end{array}$$

(8b) $$\begin{array}{cc} \; & u_{1}^{\varepsilon }\left(X,Y^{-},t\right)=u_{2}^{\varepsilon }\left(X,Y^{+},t\right),\; \end{array}$$

(8c) $$\begin{array}{cc} \; & P_{1}^{\varepsilon }\left(X,Y^{-},t\right)N\left(Y\right)=P_{2}^{\varepsilon }\left(X,Y^{+},t\right)N\left(Y\right).\; \end{array}$$

The first Piola–Kirchhoff stress tensor, $P_{\eta }^{\varepsilon }$, is related to the second Piola–Kirchhoff stress tensor, $S_{\eta }^{\varepsilon }$, through the formula

(9) $$\begin{array}{c} P_{\eta }^{\varepsilon }=F_{\eta }^{\varepsilon }S_{\eta }^{\varepsilon }, \end{array}$$

which, for nonlinear viscoelastic constituents, can be written in the form [35]

(10) $$\begin{array}{cc} S_{\eta }^{\varepsilon }\left(X,Y,t\right) & =\frac{\partial \psi_{\eta }^{\varepsilon }\left(E_{\eta }^{\varepsilon }\left(X,Y,t\right)\right)}{\partial E_{\eta }^{\varepsilon }}+\int_{-\infty }^{t}L_{\eta }^{\varepsilon }\left(X,Y,t-\tau \right):{\dot{E}}_{\eta }^{\varepsilon }\left(X,Y,\tau \right)d\tau , \end{array}$$

where $\psi_{\eta }^{\varepsilon }$ denotes the strain energy density for each constituent and $E_{\eta }^{\varepsilon }:=\frac{1}{2}\left(C_{\eta }^{\varepsilon }-I\right)$ is the Green-Lagrange strain tensor, with $C_{\eta }^{\varepsilon }:={\left(F_{\eta }^{\varepsilon }\right)}^{T}F_{\eta }^{\varepsilon }$ being the right Cauchy-Green deformation tensor. Furthermore, by embracing the terminology in [35], $L_{\eta }$ is the fourth order tensor referred to as the relaxation tensor which we assume to have left and right minor symmetries and major symmetry.

## 4. Asymptotic Homogenisation Procedure

By taking inspiration from [22,24], we propose a formal series representation for the the displacement $u_{\eta }^{\varepsilon }$ and the first Piola–Kirchhoff stress tensor $P_{\eta }^{\varepsilon }$ in powers of the smallness parameter ε, namely,

(11a) $$\begin{array}{cc} \; & u_{\eta }^{\varepsilon }\left(X,Y,t\right)=u^{\left(0\right)}\left(X,t\right)+\sum_{k=1}^{+\infty }u_{\eta }^{\left(k\right)}\left(X,Y,t\right)\varepsilon^{k}, \end{array}$$

(11b) $$\begin{array}{cc} \; & P_{\eta }^{\varepsilon }\left(X,Y,t\right)=\sum_{k=0}^{+\infty }P_{\eta }^{\left(k\right)}\left(X,Y,t\right)\varepsilon^{k}.\; \end{array}$$

The substitution of (11a) in the expression of the deformation gradient tensor (1) leads to the following power expansion

(12) $$\begin{array}{c} F_{\eta }^{\varepsilon }\left(X,Y,t\right)=\sum_{k=0}^{+\infty }F_{\eta }^{\left(k\right)}\left(X,Y,t\right)\varepsilon^{k}, \end{array}$$

where

(13a) $$\begin{array}{cc} \; & F_{\eta }^{\left(0\right)}\left(X,Y,t\right)=I+L_{c}^{-1}\left[{\mathrm{Grad}}_{X}u_{\eta }^{\left(0\right)}\left(X,t\right)+{\mathrm{Grad}}_{Y}u_{\eta }^{\left(1\right)}\left(X,Y,t\right)\right], \end{array}$$

(13b) $$\begin{array}{cc} \; & F_{\eta }^{\left(k\right)}\left(X,Y,t\right)=L_{c}^{-1}\left[{\mathrm{Grad}}_{X}u_{\eta }^{\left(k\right)}\left(X,Y,t\right)+{\mathrm{Grad}}_{Y}u_{\eta }^{\left(k+1\right)}\left(X,Y,t\right)\right]. \end{array}$$

Consequently, the series expansion of the Green-Lagrange strain tensor $E_{\eta }^{\varepsilon }$ in powers of ε is given by

(14) $$\begin{array}{c} E_{\eta }^{\varepsilon }\left(X,Y,t\right)=\sum_{k=0}^{+\infty }E_{\eta }^{\left(k\right)}\left(X,Y,t\right)\varepsilon^{k}, \end{array}$$

where, for each k=1,2,…,

(15a) $$\begin{array}{cc} \; & E_{\eta }^{\left(0\right)}\left(X,Y,t\right)=\frac{1}{2}\left[C_{\eta }^{\left(0\right)}\left(X,Y,t\right)-I\right], \end{array}$$

(15b) $$\begin{array}{cc} \; & E_{\eta }^{\left(k\right)}\left(X,Y,t\right)=\frac{1}{2}C_{\eta }^{\left(k\right)}\left(X,Y,t\right),\; \end{array}$$

and

(16) $$\begin{array}{cc} \; & C_{\eta }^{\left(k\right)}\left(X,Y,t\right)=\sum_{i=0}^{k}{F_{\eta }^{\left(i\right)}}^{T}\left(X,Y,t\right)F_{\eta }^{\left(k-i\right)}\left(X,Y,t\right). \end{array}$$

Thus, the combination and substitution of the above results in the multiscale problem defined by Equations (8a), (8b) and (8c), together with the fact that the first and second Piola–Kirchhoff stress tensors are related by the formula $P_{\eta }^{\varepsilon }=F_{\eta }^{\varepsilon }S_{\eta }^{\varepsilon }$, leads to a set of cell problems obtained by equating the coefficients of the powers of ε. Precisely, at the leading order, i.e., $\varepsilon^{0}$, we have that

(17a) $$\begin{array}{cc} \; & L_{c}^{-1}{\mathrm{Div}}_{Y}P_{\eta }^{\left(0\right)}\left(X,Y,t\right)=0,\; \end{array}$$

(17b) $$\begin{array}{cc} \; & u_{1}^{\left(1\right)}\left(X,Y^{-},t\right)=u_{2}^{\left(1\right)}\left(X,Y^{+},t\right),\; \end{array}$$

(17c) $$\begin{array}{cc} \; & P_{1}^{\left(0\right)}\left(X,Y^{-},t\right)N\left(Y\right)=P_{2}^{\left(0\right)}\left(X,Y^{+},t\right)N\left(Y\right), \end{array}$$

with

(18a) $$\begin{array}{cc} \; & P_{\eta }^{\left(0\right)}\left(X,Y,t\right)=F_{\eta }^{\left(0\right)}\left(X,Y,t\right)S_{\eta }^{\left(0\right)}\left(X,Y,t\right),\; \end{array}$$

(18b) $$\begin{array}{cc} \; & S_{\eta }^{\left(0\right)}\left(X,Y,t\right)=\frac{\partial \psi_{\eta }^{\left(0\right)}\left(E_{\eta }^{\left(0\right)}\left(X,Y,t\right)\right)}{\partial E_{\eta }^{\left(0\right)}}+\int_{-\infty }^{t}L_{\eta }^{\varepsilon }\left(X,Y,t-\tau \right):{\dot{E}}_{\eta }^{\left(0\right)}\left(X,Y,\tau \right)d\tau . \end{array}$$

Furthermore, for increasing powers of ε, namely, $\varepsilon^{k}$ with k=1,2,3,…, we can write

(19a) $$\begin{array}{cc} \; & L_{c}^{-1}\left[{\mathrm{Div}}_{X}P_{\eta }^{\left(k-1\right)}\left(X,Y,t\right)+{\mathrm{Div}}_{Y}P_{\eta }^{\left(k\right)}\left(X,Y,t\right)\right]=0, \end{array}$$

(19b) $$\begin{array}{cc} \; & u_{1}^{\left(k+1\right)}\left(X,Y^{-},t\right)=u_{2}^{\left(k+1\right)}\left(X,Y^{+},t\right),\; \end{array}$$

(19c) $$\begin{array}{cc} \; & P_{1}^{\left(k\right)}\left(X,Y^{-},t\right)N\left(Y\right)=P_{2}^{\left(k\right)}\left(X,Y^{+},t\right)N\left(Y\right),\; \end{array}$$

where

(20) $$\begin{array}{cc} \; & P_{\eta }^{\left(k\right)}\left(X,Y,t\right)=\sum_{i=0}^{k}F_{\eta }^{\left(i\right)}\left(X,Y,t\right)S_{\eta }^{\left(k-i\right)}\left(X,Y,t\right). \end{array}$$

In particular, for k=1, we have that

(21a) $$\begin{array}{cc} \; & P_{\eta }^{\left(1\right)}\left(X,Y,t\right)=F_{\eta }^{\left(0\right)}\left(X,Y,t\right)S_{\eta }^{\left(1\right)}\left(X,Y,t\right)+F_{\eta }^{\left(1\right)}\left(X,Y,t\right)S_{\eta }^{\left(0\right)}\left(X,Y,t\right),\; \end{array}$$

(21b) $$\begin{array}{cc} \; & S_{\eta }^{\left(1\right)}\left(X,Y,t\right)=\frac{\partial \psi_{\eta }^{\left(1\right)}\left(E_{\eta }^{\left(1\right)}\left(X,Y,t\right)\right)}{\partial E_{\eta }^{\left(1\right)}}+\int_{-\infty }^{t}L_{\eta }^{\varepsilon }\left(X,Y,t-\tau \right):{\dot{E}}_{\eta }^{\left(1\right)}\left(X,Y,\tau \right)d\tau . \end{array}$$

## 5. Constitutive Considerations

To exemplify the results obtained so far, we assume that the strain energy density $\psi_{\eta }^{\varepsilon }$ is of Saint–Venant type. That is, by denoting with $C_{\eta }^{\varepsilon }$ the elasticity tensor for each constituent $B_{\eta }$, with η=1,2, we have that

(22) $$\begin{array}{c} \psi_{\eta }^{\varepsilon }\left(E_{\eta }^{\varepsilon }\left(X,Y,t\right)\right)=\frac{1}{2}E_{\eta }^{\varepsilon }\left(X,Y,t\right):C_{\eta }^{\varepsilon }\left(X,Y\right):E_{\eta }^{\varepsilon }\left(X,Y,t\right), \end{array}$$

where $C_{\eta }^{\varepsilon }$ is endowed with both the left and right minor symmetries, and with the major symmetry, namely

(23) $$\begin{array}{c} {\left[C_{\eta }^{\varepsilon }\right]}_{abcd}={\left[C_{\eta }^{\varepsilon }\right]}_{bacd}={\left[C_{\eta }^{\varepsilon }\right]}_{abdc}={\left[C_{\eta }^{\varepsilon }\right]}_{cdab}, \end{array}$$

with a,b,c,d=1,2,3. The introduction of the constitutive law (23) allows to compute the derivative of $\psi_{\eta }^{\varepsilon }$ with respect to $E_{\eta }^{\varepsilon }$ in the form

$$\begin{array}{c} \frac{\partial \psi_{\eta }^{\varepsilon }\left(E_{\eta }^{\varepsilon }\left(X,Y,t\right)\right)}{\partial E_{\eta }^{\varepsilon }}=C_{\eta }^{\varepsilon }\left(X,Y\right):E_{\eta }^{\varepsilon }\left(X,Y,t\right)=\sum_{k=0}^{+\infty }C_{\eta }^{\varepsilon }\left(X,Y\right):E_{\eta }^{\left(k\right)}\left(X,Y,t\right)\varepsilon^{k}, \end{array}$$

where the terms $E_{\eta }^{\left(k\right)}$ are given in Equations (15a) and (15b). Therefore, we can individuate the coefficients in the expansion of the second Piola–Kirchhoff stress tensor as follows

(24) $$\begin{array}{c} S_{\eta }^{\left(k\right)}\left(X,Y,t\right)=C_{\eta }^{\varepsilon }\left(X,Y\right):E_{\eta }^{\left(k\right)}\left(X,Y,t\right)+\int_{-\infty }^{t}L_{\eta }^{\varepsilon }\left(X,Y,t-\tau \right):{\dot{E}}_{\eta }^{\left(k\right)}\left(X,Y,\tau \right)d\tau , \end{array}$$

which under the hypothesis of causal histories, that is by considering $E_{\eta }^{\left(k\right)}\left(X,Y,t\right)=H\left(t\right)E_{\eta }^{(k)+}\left(X,Y,t\right)$, with H(t) denoting the Heaviside step function, we have that

(25) $$\begin{array}{cc} \; & S_{\eta }^{\left(k\right)}\left(X,Y,t\right)=G_{\eta }^{\varepsilon }\left(X,Y,t\right):E_{\eta }^{(k)+}\left(X,Y,0\right)+\int_{0}^{t}G_{\eta }^{\varepsilon }\left(X,Y,t-\tau \right):{\dot{E}}_{\eta }^{(k)+}\left(X,Y,\tau \right)d\tau , \end{array}$$

where $G_{\eta }^{\varepsilon }$ is the fourth order tensor defined as $G_{\eta }^{\varepsilon }:=C_{\eta }^{\varepsilon }+L_{\eta }^{\varepsilon }$. In the following, we simplify the notation by writing $E_{\eta }^{\left(k\right)}$ instead of $E_{\eta }^{(k)+}$.

## 6. The Cell and Homogenised Problems for Infinitesimal Displacements

In order to take advantage of the elastic–viscoelastic correspondence principle (see, e.g., [12,15]), we frame the results of the previous sections in the setting of infinitesimal displacements. To this end, we introduce the infinitesimal displacement gradient $H_{\eta }\left(X,t\right)=\mathrm{Grad}\;u_{\eta }\left(X,t\right)$, where, with abuse of notation, $u_{\eta }$ denotes the infinitesimal displacement. In this context, the leading order terms of the deformation gradient tensor are given by

(26a) $$\begin{array}{cc} \; & F_{\eta }^{\left(0\right)}\left(X,Y,t\right)=I+H_{\eta }^{\left(0\right)}\left(X,Y,t\right), \end{array}$$

(26b) $$\begin{array}{cc} \; & F_{\eta }^{\left(1\right)}\left(X,Y,t\right)=H_{\eta }^{\left(1\right)}\left(X,Y,t\right),\; \end{array}$$

where $H_{\eta }^{\left(k\right)}\left(X,Y,t\right)=L_{c}^{-1}\left[{\mathrm{Grad}}_{X}u_{\eta }^{\left(k\right)}\left(X,t\right)+{\mathrm{Grad}}_{Y}u_{\eta }^{\left(k+1\right)}\left(X,Y,t\right)\right]$ for k=0,1. Thus, substituting the above results in the expansion of the right Cauchy–Green deformation tensor (16) and retaining only the linear terms, we can write

(27a) $$\begin{array}{cc} \; & C_{\eta }^{\left(0\right)}\left(X,Y,t\right)=I+H_{\eta }^{\left(0\right)}\left(X,Y,t\right)+{H_{\eta }^{\left(0\right)}}^{T}\left(X,Y,t\right), \end{array}$$

(27b) $$\begin{array}{cc} \; & C_{\eta }^{\left(1\right)}\left(X,Y,t\right)=H_{\eta }^{\left(1\right)}\left(X,Y,t\right)+{H_{\eta }^{\left(1\right)}}^{T}\left(X,Y,t\right).\; \end{array}$$

Therefore, the expressions (15a) and (15b) for the Green-Lagrange strain tensors are, within this linearised setting, given by

(28) $$\begin{array}{cc} \; & E_{\eta }^{\left(k\right)}\left(X,Y,t\right)=\mathrm{sym}(H_{\eta }^{\left(k\right)}\left(X,Y,t\right)), \end{array}$$

with k=0,1. Hence, by employing the right minor symmetry of $G_{\eta }^{\varepsilon }$, we can deduce that

(29a) $$\begin{array}{cc} \; & S_{\eta }^{\left(0\right)}\left(X,Y,t\right)=G_{\eta }^{\varepsilon }\left(X,Y,t\right):H_{\eta }^{\left(0\right)}\left(X,Y,0\right)+\int_{0}^{t}G_{\eta }^{\varepsilon }\left(X,Y,t-\tau \right):{\dot{H}}_{\eta }^{\left(0\right)}\left(X,Y,\tau \right)d\tau , \end{array}$$

(29b) $$\begin{array}{cc} \; & S_{\eta }^{\left(1\right)}\left(X,Y,t\right)=G_{\eta }^{\varepsilon }\left(X,Y,t\right):H_{\eta }^{\left(1\right)}\left(X,Y,0\right)+\int_{0}^{t}G_{\eta }^{\varepsilon }\left(X,Y,t-\tau \right):{\dot{H}}_{\eta }^{\left(1\right)}\left(X,Y,\tau \right)d\tau . \end{array}$$

Thus, after substitution in (18a) and (21a), and retaining only the linear terms, we can deduce that

(30a) $$\begin{array}{cc} \; & P_{\eta }^{\left(0\right)}\left(X,Y,t\right)=G_{\eta }^{\varepsilon }\left(X,Y,t\right):H_{\eta }^{\left(0\right)}\left(X,Y,0\right)+\int_{0}^{t}G_{\eta }^{\varepsilon }\left(X,Y,t-\tau \right):{\dot{H}}_{\eta }^{\left(0\right)}\left(X,Y,\tau \right)d\tau , \end{array}$$

(30b) $$\begin{array}{cc} \; & P_{\eta }^{\left(1\right)}\left(X,Y,t\right)=G_{\eta }^{\varepsilon }\left(X,Y,t\right):H_{\eta }^{\left(1\right)}\left(X,Y,0\right)+\int_{0}^{t}G_{\eta }^{\varepsilon }\left(X,Y,t-\tau \right):{\dot{H}}_{\eta }^{\left(1\right)}\left(X,Y,\tau \right)d\tau . \end{array}$$

### 6.1. The First Cell Problem

We notice that the integral in (30a) represents the convolution of $G_{\eta }^{\varepsilon }$ and ${\dot{H}}_{\eta }$, so that the application of the Laplace transform leads to

(31) $$\begin{array}{c} {\tilde{P}}_{\eta }^{\left(0\right)}\left(X,Y,s\right)=s{\tilde{G}}_{\eta }^{\varepsilon }\left(X,Y,s\right):{\tilde{H}}_{\eta }^{\left(0\right)}\left(X,Y,s\right)={\hat{G}}_{\eta }^{\varepsilon }\left(X,Y,s\right):{\tilde{H}}_{\eta }^{\left(0\right)}\left(X,Y,s\right), \end{array}$$

where *s* is a complex variable dual to time and ${\hat{G}}_{\eta }^{\varepsilon }$ denotes the Laplace–Carson transform of $G_{\eta }^{\varepsilon }$, that is, ${\hat{G}}_{\eta }^{\varepsilon }=s{\tilde{G}}_{\eta }^{\varepsilon }=C_{\eta }^{\varepsilon }+s{\tilde{L}}_{\eta }^{\varepsilon }$ with ${\tilde{G}}_{\eta }^{\varepsilon }$ and ${\tilde{L}}_{\eta }^{\varepsilon }$ being the Laplace transform of $G_{\eta }$ and $L_{\eta }^{\varepsilon }$. Since the elasticity tensor, $C_{\eta }^{\varepsilon }$, is time independent, we can also write ${\hat{G}}_{\eta }^{\varepsilon }={\hat{C}}_{\eta }^{\varepsilon }+{\hat{L}}_{\eta }^{\varepsilon }$. Therefore, by introducing the ansatz

(32) $$\begin{array}{c} {\tilde{u}}_{\eta }^{\left(1\right)}\left(X,Y,s\right)={\tilde{\chi }}_{\eta }\left(X,Y,s\right):{\mathrm{Grad}}_{X}{\tilde{u}}_{\eta }^{\left(0\right)}\left(X,s\right)+{\tilde{\omega }}_{\eta }\left(X,s\right), \end{array}$$

where ${\tilde{\chi }}_{\eta }$ represents the Laplace transform of the *Y*-periodic, third order tensor $\chi_{\eta }$ and ${\tilde{\omega }}_{\eta }$ is a *Y*-constant vector field, we find, from Equation (17a), the *cell* or *local problem*

(33a) $$\begin{array}{cc} \; & L_{c}^{-1}{\mathrm{Div}}_{Y}({\hat{G}}_{\eta }^{\varepsilon }\left(X,Y,s\right)\;+{\hat{G}}_{\eta }^{\varepsilon }\left(X,Y,s\right):{\mathrm{Grad}}_{Y}{\tilde{\chi }}_{\eta }^{T}\left(X,Y,s\right))=0, \end{array}$$

(33b) $$\begin{array}{cc} \; & {\tilde{u}}_{1}^{\left(1\right)}\left(X,Y^{-},s\right)={\tilde{u}}_{2}^{\left(1\right)}\left(X,Y^{+},s\right),\; \end{array}$$

(33c) $$\begin{array}{cc} \; & [{\hat{G}}_{1}^{\varepsilon }\left(X,Y^{-},s\right)+{\hat{G}}_{1}^{\varepsilon }\left(X,Y^{-},s\right):{\mathrm{Grad}}_{Y}{\tilde{\chi }}_{1}^{T}\left(X,Y^{-},s\right)]N\left(Y\right)=\;\\ \; & =[{\hat{G}}_{2}^{\varepsilon }\left(X,Y^{+},s\right)+{\hat{G}}_{2}^{\varepsilon }\left(X,Y^{+},s\right):{\mathrm{Grad}}_{Y}{\tilde{\chi }}_{2}^{T}\left(X,Y^{+},s\right)]N\left(Y\right). \end{array}$$

We notice that Equations (33a), (33b) and (33c) determine the classical cell problem resulting from the use of the asymptotic homogenisation in linear viscoelasticity (see, for instance, [10,11,15]). Following the discussion in [24], in Equation (33a), the components of ${\mathrm{Grad}}_{Y}{\tilde{\chi }}_{\eta }^{T}$ are

(34) $$\begin{array}{c} {\left[{\mathrm{Grad}}_{Y},{\tilde{\chi }}_{\eta }^{T}\right]}_{abcd}:=\frac{\partial {\left[{\tilde{\chi }}_{\eta }^{T}\right]}_{cda}}{\partial Y_{b}}=\frac{\partial {\left[{\tilde{\chi }}_{\eta }\right]}_{acd}}{\partial Y_{b}}. \end{array}$$

### 6.2. The Second Cell Problem

The second cell problem is given by Equations (19a), (19b) and (19c) for k=1. In view of the results obtained in the previous sections, the first equation of the second cell problem, that is Equation (19a), can be equivalently rewritten as

(35) $$\begin{array}{cc} \; & L_{c}^{-1}[{\mathrm{Div}}_{X}({\hat{G}}_{\eta }^{\varepsilon }\left(X,Y,s\right):{\tilde{H}}_{\eta }^{\left(0\right)}\left(X,Y,s\right))+{\mathrm{Div}}_{Y}({\hat{G}}_{\eta }^{\varepsilon }\left(X,Y,s\right):{\tilde{H}}_{\eta }^{\left(1\right)}\left(X,Y,s\right))]=0. \end{array}$$

Thus, by introducing, for a generic field $\Phi_{\eta }^{\varepsilon }\left(X,Y,s\right)$, the integral operators ${\langle •\rangle }_{Y_{\eta }}$ over the portion $Y_{\eta }$ of the unit cell Y, namely

(36) $$\begin{array}{c} {\langle \Phi_{\eta }^{\varepsilon }\rangle }_{Y_{\eta }}\left(X,s\right):=\frac{1}{\left|Y\right|}\int_{Y_{\eta }}\Phi_{\eta }^{\varepsilon }\left(X,Y,s\right)dV\left(Y\right), \end{array}$$

where |Y| represents the measure of the cell, and applying it to Equation (35), we can deduce that

(37) $$\begin{array}{cc} \; & L_{c}^{-1}[{\mathrm{Div}}_{X}{\langle {\hat{G}}_{\eta }^{\varepsilon }\left(X,Y,s\right):{\tilde{H}}_{\eta }^{\left(0\right)}\left(X,Y,s\right)\rangle }_{Y_{\eta }}+{\langle {\mathrm{Div}}_{Y}({\hat{G}}_{\eta }^{\varepsilon }\left(X,Y,s\right):{\tilde{H}}_{\eta }^{\left(1\right)}\left(X,Y,s\right))\rangle }_{Y_{\eta }}]=0, \end{array}$$

where we have taken into account the consideration of macroscopic uniformity. Therefore, since the second addend at the left-hand side of (37) reduces to the zero vector because of the assumption of local periodicity and the ideal contact conditions at the interface (refer to [28] for further details), we find the homogenised equation

(38) $$\begin{array}{cc} \; & L_{c}^{-1}{\mathrm{Div}}_{X}({\hat{G}}_{\mathrm{eff}}\left(X,s\right):{\mathrm{Grad}}_{X}{\tilde{u}}^{\left(0\right)}\left(X,s\right))=0, \end{array}$$

where the effective coefficient ${\hat{G}}_{\mathrm{eff}}$ is defined as

(39) $$\begin{array}{c} {\hat{G}}_{\mathrm{eff}}\left(X,s\right)=\sum_{\eta =1}^{2}{\langle {\hat{G}}_{\eta }^{\varepsilon }\left(X,Y,s\right)+{\hat{G}}_{\eta }^{\varepsilon }\left(X,Y,s\right):{\mathrm{Grad}}_{Y}{\tilde{\chi }}_{\eta }^{T}\left(X,Y,s\right)\rangle }_{Y_{\eta }}. \end{array}$$

## 7. Uniaxially Fibre-Reinforced Composites

**Figure 2:**
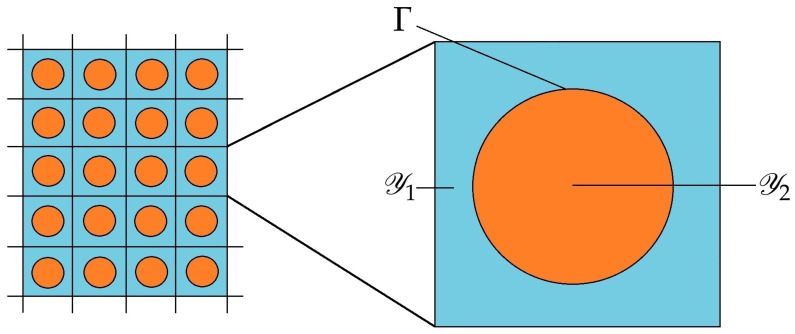
Schematic of the cross section of the fibre reinforced composite.

We consider the case of a fibre reinforced composite with uniaxially oriented cylindrical fibres oriented in the direction specified by $i_{3}$, where ${\left\{i_{k}\right\}}_{k=1}^{3}$ denotes the standard Cartesian vector basis. In this framework, we represent the cross section of the microstructure as a matrix with evenly distributed circular inclusions and, especially, we choose the unit cell to be characterised by a square with a single inclusion $Y_{2}$. That is, the portion of the cell representing the matrix is $Y_{1}=Y\backslash Y_{2}$ as shown in Figure 2.

Within this context, the unknowns and fields of interest depend only on the macroscale spatial coordinates $X_{1}$ and $X_{2}$, on the microscale coordinates $Y_{1}$ and $Y_{2}$, and on *s*. Consequently, we have that the leading order-term of the infinitesimal displacement gradient tensor, $H_{\eta }^{\left(0\right)}$, can be additively decomposed as follows

(40) $$\begin{array}{c} \left[{\tilde{H}}_{\eta }^{\left(0\right)}\right]=[\begin{array}{ccc} {\left[{\tilde{H}}_{\eta }^{\left(0\right)}\right]}_{11} & {\left[{\tilde{H}}_{\eta }^{\left(0\right)}\right]}_{12} & 0\\ {\left[{\tilde{H}}_{\eta }^{\left(0\right)}\right]}_{21} & {\left[{\tilde{H}}_{\eta }^{\left(0\right)}\right]}_{22} & 0\\ 0 & 0 & 0 \end{array}]+[\begin{array}{ccc} 0 & 0 & 0\\ 0 & 0 & 0\\ {\left[{\tilde{H}}_{\eta }^{\left(0\right)}\right]}_{31} & {\left[{\tilde{H}}_{\eta }^{\left(0\right)}\right]}_{32} & 0 \end{array}], \end{array}$$

where the decomposition (40), together with the linearity of the problem at hand and the superposition principle, permit us to split the cell problem (33a) into an in-plane and an anti-plane contribution. Here, we focus on solving only the anti-plane cell problem, which means that we work only with the second addend in (40), so that the anti-plane shear stresses are

(41) $$\begin{array}{c} {\left[{\tilde{P}}_{\eta \;a}^{\left(0\right)}\right]}_{ab}={\left[{\hat{G}}_{\eta }^{\varepsilon }\right]}_{ab31}{\left[{\tilde{H}}_{\eta }^{\left(0\right)}\right]}_{31}+{\left[{\hat{G}}_{\eta }^{\varepsilon }\right]}_{ab32}{\left[{\tilde{H}}_{\eta }^{\left(0\right)}\right]}_{32}, \end{array}$$

where the subindex “a” in ${\tilde{P}}_{\eta \;a}^{\left(0\right)}$ is used to specify that only the anti-plane contribution is being taken into account.

### 7.1. Anti-Plane Cell Problems and Effective Coefficients

For generic monoclinic constituents, the relevant constitutive relation is [36,37],

(42) $$\begin{array}{cc} \; & {\left[{\tilde{P}}_{\eta \;a}^{\left(0\right)}\right]}_{3b}={\left[{\hat{G}}_{\eta }^{\varepsilon }\right]}_{3b31}{\left[{\tilde{H}}_{\eta }^{\left(0\right)}\right]}_{31}+{\left[{\hat{G}}_{\eta }^{\varepsilon }\right]}_{3b32}{\left[{\tilde{H}}_{\eta }^{\left(0\right)}\right]}_{32}, \end{array}$$

with b=1,2. Thus, because of the above considerations, the relevant components of the effective coefficient associated with the anti-plane problem are given by

(43) $$\begin{array}{c} {\left[{\hat{G}}_{\mathrm{eff}}\left(X,s\right)\right]}_{3b3d}=\sum_{\eta =1}^{2}{\langle {\left[{\hat{G}}_{\eta }^{\varepsilon }\left(X,Y,s\right)\right]}_{3b3d}+{\left[{\hat{G}}_{\eta }^{\varepsilon }\left(X,Y,s\right)\right]}_{3b3q}\frac{\partial {\left[{\tilde{\chi }}_{\eta }\left(X,Y,s\right)\right]}_{33d}}{\partial Y_{q}}\rangle }_{Y_{\eta }}, \end{array}$$

with b,d=1,2, which can be found by solving the cell problems,

(44a) $$\begin{array}{cc} \; & L_{c}^{-1}\frac{\partial }{\partial Y^{b}}({\left[{\hat{G}}_{\eta }^{\varepsilon }\left(X,Y,s\right)\right]}_{3b3d}+{\left[{\hat{G}}_{\eta }^{\varepsilon }\left(X,Y,s\right)\right]}_{3b3q}\frac{\partial {\left[{\tilde{\chi }}_{\eta }\left(X,Y,s\right)\right]}_{33d}}{\partial Y_{q}})=0, \end{array}$$

(44b) $$\begin{array}{cc} \; & {\left[{\tilde{\chi }}_{1}\left(X,Y^{-},s\right)\right]}_{33d}={\left[{\tilde{\chi }}_{2}\left(X,Y^{+},s\right)\right]}_{33d},\;\\ \; & ({\left[{\hat{G}}_{1}^{\varepsilon }\left(X,Y^{-},s\right)\right]}_{3b3d}+{\left[{\hat{G}}_{1}^{\varepsilon }\left(X,Y^{+},s\right)\right]}_{3b3q}\frac{\partial {\left[{\tilde{\chi }}_{1}\left(X,Y^{-},s\right)\right]}_{33d}}{\partial Y_{q}})N_{b}\; \end{array}$$

(44c) $$\begin{array}{cc} \; & \;=({\left[{\hat{G}}_{2}^{\varepsilon }\left(X,Y^{+},s\right)\right]}_{3b3d}+{\left[{\hat{G}}_{2}^{\varepsilon }\left(X,Y^{+},s\right)\right]}_{3b3q}\frac{\partial {\left[{\tilde{\chi }}_{2}\left(X,Y^{+},s\right)\right]}_{33d}}{\partial Y_{q}})N_{b}.\; \end{array}$$

Equations (44a), (44b) and (44c), for d=1,2, represent two anti-plane cell problems and each one of them help to compute specific effective coefficients (see, e.g., [16]). Specifically, for d=1, the cell problem provides ${\left[{\hat{G}}_{\mathrm{eff}}\right]}_{3131}$ and ${\left[{\hat{G}}_{\mathrm{eff}}\right]}_{3132}$, while for d=2, it allows to compute ${\left[{\hat{G}}_{\mathrm{eff}}\right]}_{3231}$ and ${\left[{\hat{G}}_{\mathrm{eff}}\right]}_{3232}$. Therefore, by further considering that the tensor ${\hat{G}}_{\eta }^{\varepsilon }$ has tetragonal symmetry, so that ${\left[{\hat{G}}_{\eta }^{\varepsilon }\right]}_{3131}={\left[{\hat{G}}_{\eta }^{\varepsilon }\right]}_{3232}$ and ${\left[{\hat{G}}_{\eta }^{\varepsilon }\right]}_{3132}={\left[{\hat{G}}_{\eta }^{\varepsilon }\right]}_{3231}=0$ [15], and that the fourth-order tensors ${\hat{G}}_{\eta }^{\varepsilon }$ are of the form ${\hat{G}}_{\eta }^{\varepsilon }\left(X,Y,s\right)={\hat{G}}_{\eta }^{\varepsilon }\left(s\right)$ (i.e., we assume that the material properties of each constituent depend only on *s*), the expressions for the effective coefficients corresponding to the anti-plane problem can be written as

(45a) $$\begin{array}{cc} \; & {\left[{\hat{G}}_{\mathrm{eff}}\left(s\right)\right]}_{3131}=\sum_{\eta =1}^{2}{\langle {\left[{\hat{G}}_{\eta }^{\varepsilon }\left(s\right)\right]}_{3131}+{\left[{\hat{G}}_{\eta }^{\varepsilon }\left(s\right)\right]}_{3131}\frac{\partial {\left[{\tilde{\chi }}_{\eta }\left(Y,s\right)\right]}_{331}}{\partial Y_{1}}\rangle }_{Y_{\eta }}, \end{array}$$

(45b) $$\begin{array}{cc} \; & {\left[{\hat{G}}_{\mathrm{eff}}\left(s\right)\right]}_{3132}=\sum_{\eta =1}^{2}{\langle {\left[{\hat{G}}_{\eta }^{\varepsilon }\left(s\right)\right]}_{3131}\frac{\partial {\left[{\tilde{\chi }}_{\eta }\left(Y,s\right)\right]}_{332}}{\partial Y_{1}}\rangle }_{Y_{\eta }},\; \end{array}$$

(45c) $$\begin{array}{cc} \; & {\left[{\hat{G}}_{\mathrm{eff}}\left(s\right)\right]}_{3231}=\sum_{\eta =1}^{2}{\langle {\left[{\hat{G}}_{\eta }^{\varepsilon }\left(s\right)\right]}_{3232}\frac{\partial {\left[{\tilde{\chi }}_{\eta }\left(Y,s\right)\right]}_{331}}{\partial Y_{2}}\rangle }_{Y_{\eta }},\; \end{array}$$

(45d) $$\begin{array}{cc} \; & {\left[{\hat{G}}_{\mathrm{eff}}\left(s\right)\right]}_{3232}=\sum_{\eta =1}^{2}{\langle {\left[{\hat{G}}_{\eta }^{\varepsilon }\left(s\right)\right]}_{3232}+{\left[{\hat{G}}_{\eta }^{\varepsilon }\left(s\right)\right]}_{3232}\frac{\partial {\left[{\tilde{\chi }}_{\eta }\left(Y,s\right)\right]}_{332}}{\partial Y_{2}}\rangle }_{Y_{\eta }}, \end{array}$$

and the cell problem reduces to

(46a) $$\begin{array}{cc} \; & L_{c}^{-1}{\left[{\hat{G}}_{\eta }^{\varepsilon }\left(s\right)\right]}_{3131}\frac{\partial^{2}{\left[{\tilde{\chi }}_{\eta }\left(Y,s\right)\right]}_{33d}}{\partial Y_{b}\partial Y_{b}}=0,\; \end{array}$$

(46b) $$\begin{array}{cc} \; & {\left[{\tilde{\chi }}_{1}\left(Y^{-},s\right)\right]}_{33d}={\left[{\tilde{\chi }}_{2}\left(Y^{+},s\right)\right]}_{33d},\;\\ \; & ({\left[{\hat{G}}_{1}^{\varepsilon }\left(s\right)\right]}_{3131}\frac{\partial {\left[{\tilde{\chi }}_{1}\left(Y^{-},s\right)\right]}_{33d}}{\partial Y_{b}}-{\left[{\hat{G}}_{2}^{\varepsilon }\left(s\right)\right]}_{3131}\frac{\partial {\left[{\tilde{\chi }}_{2}\left(Y^{+},s\right)\right]}_{33d}}{\partial Y_{b}})N_{b} \end{array}$$

(46c) $$\begin{array}{cc} \; & \;=-({\left[{\hat{G}}_{1}^{\varepsilon }\left(s\right)\right]}_{3b3d}-{\left[{\hat{G}}_{2}^{\varepsilon }\left(s\right)\right]}_{3b3d})N_{b}.\; \end{array}$$

### 7.2. Solution of the Anti-Plane Cell Problem

To find the solution of the the cell problem (46a), (46b) and (46c), we adhere to the procedure discussed in [15] for a square arrangement of cells with an embedded circular inclusion. The methodology, which is based on the the theory of harmonic functions and the Kolosov–Muskhelishvili complex potentials [38] has been extensively investigated in different scenarios (see, for instance, [16,39]). Here, we just report the main steps and refer the Reader to [15,16]. Therefore, we set

(47) $$\begin{array}{cc} \; & {\tilde{\chi }}_{\eta }^{331}\left(Y,s\right)=Re(\varphi_{\eta }^{1}\left(Z,s\right))\;\mathrm{and}\;{\tilde{\chi }}_{\eta }^{332}\left(Y,s\right)=Im(\varphi_{\eta }^{2}\left(Z,s\right)), \end{array}$$

where, for d=1,2, the complex potentials can be written as

(48) $$\begin{array}{cc} \; & \varphi_{1}^{d}\left(Z,s\right)={\sum_{l=1}^{\infty }}^{o}(a_{l}^{d}\left(s\right)Z^{-l}-A_{l}^{d}\left(s\right)Z^{l})\;\mathrm{and}\;\varphi_{2}^{d}\left(Z,s\right)={\sum_{l=1}^{\infty }}^{o}c_{l}^{d}\left(s\right)Z^{l}. \end{array}$$

In particular, the symbol “o” indicates that the sum is performed over odd indices, $a_{l}^{d}\left(s\right)$ and $c_{l}^{d}\left(s\right)$ are complex coefficients and $A_{l}^{d}\left(s\right):={\sum_{m=1}^{\infty }}^{o}m\Lambda_{ml}a_{m}^{d}\left(s\right)$. In the latter expression, the elements of the matrix Λ are defined as

(49) $$\begin{array}{c} \Lambda_{ml}=\{\begin{array}{cc} {}[S_{m+l}\left(m+l-1\right)!]/\left(m!l!\right) & \mathrm{if}\;m,l>1\\ {\left(-1\right)}^{d+1}\pi & \mathrm{if}\;m=l=1 \end{array}\}, \end{array}$$

where the term $S_{m+l}$ denotes the reticular sums, namely $S_{m+l}=\sum_{w\in L^{*}}w^{-(m+l)}$ for $w=rw_{1}+sw_{2}$ with r,s∈Z and $L^{*}$ representing the lattice excluding w=0 (see [17] for further details).

The substitution of the above expressions in the interface condition (46b) leads to the following algebraic equations involving the complex coefficients $a_{l}^{d}$ and $c_{l}^{d}$

(50) $$\begin{array}{c} {\left(-1\right)}^{d+1}{\bar{a}}_{l}^{d}\left(s\right)R^{-l}-A_{l}^{d}\left(s\right)R^{l}=c_{l}^{d}\left(s\right)R^{l}, \end{array}$$

where d=1,2, *R* is the radius of the circular inclusion and $Z=Re^{i\theta }$, with θ∈[0,2π], characterises the interface between the inclusion and the matrix. Furthermore, following similar procedure on the second interface condition (46c) yields

(51) $$\begin{array}{c} {\bar{a}}_{l}^{d}\left(s\right)R^{-l}+{\left(-1\right)}^{d+1}\xi \left(s\right)A_{l}^{d}\left(s\right)R^{l}={\left(-1\right)}^{d+1}\xi \left(s\right)R^{l}\delta_{1l}, \end{array}$$

where ξ(s) is given by the expression

(52) $$\begin{array}{c} \xi \left(s\right)=\frac{{\left[{\hat{G}}_{1}^{\varepsilon }\left(s\right)\right]}_{3131}-{\left[{\hat{G}}_{2}^{\varepsilon }\left(s\right)\right]}_{3131}}{{\left[{\hat{G}}_{1}^{\varepsilon }\left(s\right)\right]}_{3131}+{\left[{\hat{G}}_{2}^{\varepsilon }\left(s\right)\right]}_{3131}}. \end{array}$$

In particular, rescaling the problem (51) with $a_{l}^{d}\left(s\right)=\left[b_{l}^{d}\left(s\right)R^{l}\right]/\sqrt{l}$ and considering the real and imaginary parts, the system of linear Equation (51) can be equivalently rewritten in the form

(53a) $$\begin{array}{ll} \; & ℜ\left(b_{l}^{d}\left(s\right)\right)+{\left(-1\right)}^{d+1}{\sum_{m=1}^{\infty }}^{o}\left\{\left[ℜ\left(\xi \left(s\right)\right)ℜ\left(W_{ml}^{d}\right)-ℑ\left(\xi \left(s\right)\right)ℑ\left(W_{ml}^{d}\right)\right]ℜ\left(b_{m}^{d}\left(s\right)\right)\right\}\\ \; & \;+{\left(-1\right)}^{d+1}{\sum_{m=1}^{\infty }}^{o}\left\{\left[-ℜ\left(\xi \left(s\right)\right)ℑ\left(W_{ml}^{d}\right)-ℑ\left(\xi (s)\right)ℜ\left(W_{ml}^{d}\right)\right]ℑ\left(b_{m}^{d}\left(s\right)\right)\right\}\;\\ \; & \;={\left(-1\right)}^{d+1}R^{l}\sqrt{l}ℜ\left(\xi \left(s\right)\right)\delta_{1l},\; \end{array}$$

(53b) $$\begin{array}{cc} \; & ℑ\left(b_{l}^{d}\left(s\right)\right)+{\left(-1\right)}^{d+1}{\sum_{m=1}^{\infty }}^{o}\left\{\left[-ℜ\left(\xi \left(s\right)\right)ℑ\left(W_{ml}^{d}\right)-ℑ\left(\xi \left(s\right)\right)ℜ\left(W_{ml}^{d}\right)\right]ℜ\left(b_{m}^{d}\left(s\right)\right)\right\}\\ \; & \;+{\left(-1\right)}^{d+1}{\sum_{m=1}^{\infty }}^{o}\left\{\left[-ℜ\left(\xi \left(s\right)\right)ℜ\left(W_{ml}^{d}\right)+ℑ\left(\xi \left(s\right)\right)ℑ\left(W_{ml}^{d}\right)\right]ℑ\left(b_{m}^{d}\left(s\right)\right)\right\}\\ \; & \;=-{\left(-1\right)}^{d+1}R^{l}\sqrt{l}ℑ\left(\xi \left(s\right)\right)\delta_{1l}, \end{array}$$

where ℜ(·) and ℑ(·) extract the real and imaginary parts of the complex quantities they are applied to. We notice that, Equations (53a) and (53b) can be represented in the equivalent form

(54) $$\begin{array}{c} \;[\begin{array}{c} b_{r}^{d}\left(s\right)\\ b_{i}^{d}\left(s\right) \end{array}]={\left([\begin{array}{cc} I & O\\ O & I \end{array}]+[\begin{array}{cc} \xi_{r}\left(s\right)W_{r}^{d}-\xi_{i}\left(s\right)W_{i}^{d} & -\xi_{r}\left(s\right)W_{i}^{d}-\xi_{i}\left(s\right)W_{r}^{d}\\ -\xi_{r}\left(s\right)W_{i}^{d}-\xi_{i}\left(s\right)W_{r}^{d} & -\xi_{r}\left(s\right)W_{r}^{d}+\xi_{i}\left(s\right)W_{i}^{d} \end{array}]\right)}^{-1}[\begin{array}{c} V_{r}^{d}\left(s\right)\\ V_{i}^{d}\left(s\right) \end{array}], \end{array}$$

where we have introduced the notations $\xi_{r}$ and $\xi_{i}$ for the real and imaginary parts of ξ, and

(55a) $$\begin{array}{cc} \; & b_{r}^{d}\left(s\right)=ℜ{\left(b_{1}^{d}\left(s\right),b_{3}^{d}\left(s\right),\ldots \right)}^{T}\;\mathrm{and}\;b_{i}^{d}\left(s\right)=ℑ{\left(b_{1}^{d}\left(s\right),b_{3}^{d}\left(s\right),\ldots \right)}^{T}, \end{array}$$

(55b) $$\begin{array}{cc} \; & {\left[W_{r}^{d}\right]}_{ml}=ℜ(W_{ml}^{d})\;\mathrm{and}\;{\left[W_{i}^{d}\right]}_{ml}=ℑ(W_{ml}^{d}),\; \end{array}$$

(55c) $$\begin{array}{cc} \; & V_{r}^{d}\left(s\right)=ℜ{\left({\left(-1\right)}^{d+1}R\xi \left(s\right),0,0,\ldots \right)}^{T},\; \end{array}$$

(55d) $$\begin{array}{cc} \; & V_{i}^{d}\left(s\right)=ℑ{\left(-{\left(-1\right)}^{d+1}R\xi \left(s\right),0,0,\ldots \right)}^{T}.\; \end{array}$$

Furthermore, we denote with I and O the infinite identity and zero matrices, respectively. We remark that in the limit case of linear elastic constituents, Equation (54) reduces to the one provided in [24].

### 7.3. Effective Coefficients

To find closed-form expressions for the effective coefficients, we follow, with slight modifications, the approach depicted in [16,17]. We notice that, for the problem at hand, the effective coefficients given in (43) can be specified as

(56) $$\begin{array}{cc} {\left[{\hat{G}}_{\mathrm{eff}}\left(s\right)\right]}_{3b3d} & =\frac{1}{\left|Y\right|}\int_{Y_{1}}\left({\left[{\hat{G}}_{1}^{\varepsilon }\left(s\right)\right]}_{3b3d}+{\left[{\hat{G}}_{1}^{\varepsilon }\left(s\right)\right]}_{3b3q}\frac{\partial {\left[{\tilde{\chi }}_{1}\left(Y,s\right)\right]}_{33d}}{\partial Y_{q}}\right)dA(Y)\;\\ \; & \;+\frac{1}{\left|Y\right|}\int_{Y_{2}}\left({\left[{\hat{G}}_{2}^{\varepsilon }\left(s\right)\right]}_{3b3d}+{\left[{\hat{G}}_{2}^{\varepsilon }\left(s\right)\right]}_{3b3q}\frac{\partial {\left[{\tilde{\chi }}_{2}\left(Y,s\right)\right]}_{33d}}{\partial Y_{q}}\right)dA\left(Y\right), \end{array}$$

so that, due to the local periodicity of ${\tilde{\chi }}_{\eta }$, we can write

(57a) $$\begin{array}{cc} \; & {\left[{\hat{G}}_{\mathrm{eff}}\left(s\right)\right]}_{3131}=\sum_{\eta =1}^{2}{\langle {\left[{\hat{G}}_{\eta }^{\varepsilon }\left(s\right)\right]}_{3131}\rangle }_{Y_{\eta }}+\frac{{\left[{\hat{G}}_{1}^{\varepsilon }\left(s\right)\right]}_{3131}}{\left|Y\right|}\int_{\Gamma_{Y}}\left({\left[{\tilde{\chi }}_{2}\left(Y,s\right)\right]}_{331}-{\left[{\tilde{\chi }}_{1}\left(Y,s\right)\right]}_{331}\right)dY_{2}, \end{array}$$

(57b) $$\begin{array}{cc} \; & {\left[{\hat{G}}_{\mathrm{eff}}\left(s\right)\right]}_{3232}=\sum_{\eta =1}^{2}{\langle {\left[{\hat{G}}_{\eta }^{\varepsilon }\left(s\right)\right]}_{3232}\rangle }_{Y_{\eta }}+\frac{{\left[{\hat{G}}_{1}^{\varepsilon }\left(s\right)\right]}_{3232}}{\left|Y\right|}\int_{\Gamma_{Y}}\left({\left[{\tilde{\chi }}_{2}\left(Y,s\right)\right]}_{332}-{\left[{\tilde{\chi }}_{1}\left(Y,s\right)\right]}_{332}\right)dY_{1}, \end{array}$$

and

(58a) $$\begin{array}{cc} \; & {\left[{\hat{G}}_{\mathrm{eff}}\left(s\right)\right]}_{3132}=\frac{{\left[{\hat{G}}_{1}^{\varepsilon }\left(s\right)\right]}_{3131}}{\left|Y\right|}\int_{\Gamma_{Y}}\left({\left[{\tilde{\chi }}_{2}\left(Y,s\right)\right]}_{332}-{\left[{\tilde{\chi }}_{1}\left(Y,s\right)\right]}_{332}\right)dY_{2}, \end{array}$$

(58b) $$\begin{array}{cc} \; & {\left[{\hat{G}}_{\mathrm{eff}}\left(s\right)\right]}_{3231}=\frac{{\left[{\hat{G}}_{1}^{\varepsilon }\left(s\right)\right]}_{3232}}{\left|Y\right|}\int_{\Gamma_{Y}}\left({\left[{\tilde{\chi }}_{2}\left(Y,s\right)\right]}_{331}-{\left[{\tilde{\chi }}_{1}\left(Y,s\right)\right]}_{331}\right)dY_{1}. \end{array}$$

Thus, after substitution of (47) in the above expressions, it is possible to write

(59a) $$\begin{array}{cc} \; & {\left[{\hat{G}}_{\mathrm{eff}}\left(s\right)\right]}_{3131}-i{\left[{\hat{G}}_{\mathrm{eff}}\left(s\right)\right]}_{3231}={\left[{\hat{G}}_{1}^{\varepsilon }\left(s\right)\right]}_{3131}(1-2\pi {\bar{a}}_{1}^{1}\left(s\right)), \end{array}$$

(59b) $$\begin{array}{cc} \; & {\left[{\hat{G}}_{\mathrm{eff}}\left(s\right)\right]}_{3132}-i{\left[{\hat{G}}_{\mathrm{eff}}\left(s\right)\right]}_{3232}=-{\left[{\hat{G}}_{1}^{\varepsilon }\left(s\right)\right]}_{3232}(1+2\pi {\bar{a}}_{1}^{2}\left(s\right))i. \end{array}$$

In the case of tetragonal symmetry of the fourth-order tensor ${\hat{G}}_{\eta }^{\varepsilon }$, we know that ${\left[{\hat{G}}_{\mathrm{eff}}\right]}_{3132}={\left[{\hat{G}}_{\mathrm{eff}}\right]}_{3231}=0$ (see, for instance, [15]). Consequently, (59a) and (59b) reduce to

(60a) $$\begin{array}{cc} \; & {\left[{\hat{G}}_{\mathrm{eff}}\left(s\right)\right]}_{3131}={\left[{\hat{G}}_{1}^{\varepsilon }\left(s\right)\right]}_{3131}(1-2\pi {\bar{a}}_{1}^{1}\left(s\right)), \end{array}$$

(60b) $$\begin{array}{cc} \; & {\left[{\hat{G}}_{\mathrm{eff}}\left(s\right)\right]}_{3232}={\left[{\hat{G}}_{1}^{\varepsilon }\left(s\right)\right]}_{3131}(1+2\pi {\bar{a}}_{1}^{2}\left(s\right)). \end{array}$$

## 8. Benchmark Problems

Let us consider that both constituents $B_{1}$ and $B_{2}$ are isotropic. In the present framework, this consideration implies that the elastic and relaxation tensors have the following representations

(61a) $$\begin{array}{cc} \; & C_{\eta }^{\varepsilon }\left(X,Y\right)=3\kappa_{\eta }^{e}K+2\mu_{\eta }^{e}M, \end{array}$$

(61b) $$\begin{array}{cc} \; & L_{\eta }^{\varepsilon }\left(X,Y,t\right)=3\kappa_{\eta }^{v}\left(t\right)K+2\mu_{\eta }^{v}\left(t\right)M, \end{array}$$

where $\kappa_{\eta }^{e}$ and $\mu_{\eta }^{e}$ represent the elastic bulk modulus and the second Lamé’s parameter, respectively, and $\kappa_{\eta }^{v}$ and $\mu_{\eta }^{v}$ are memory functions which need to be constitutively chosen. Furthermore, the fourth order tensor $K:=\frac{1}{3}I⊗I$ extracts the spherical part of a second-order tensor, while $M:=\frac{1}{2}\left[I\underline{⊗}I+I\bar{⊗}I\right]-K$ extracts its deviatoric part. Thus, recalling that ${\hat{G}}_{\eta }^{\varepsilon }={\hat{C}}_{\eta }^{\varepsilon }+{\hat{L}}_{\eta }^{\varepsilon }$, we can deduce that

(62a) $$\begin{array}{cc} \; & {\left[{\hat{G}}_{\mathrm{eff}}\left(s\right)\right]}_{3131}=[\mu_{1}^{e}+{\hat{\mu }}_{1}^{v}\left(s\right)][1-2\pi {\bar{a}}_{1}^{1}\left(s\right)], \end{array}$$

(62b) $$\begin{array}{cc} \; & {\left[{\hat{G}}_{\mathrm{eff}}\left(s\right)\right]}_{3232}=[\mu_{1}^{e}+{\hat{\mu }}_{1}^{v}\left(s\right)][1+2\pi {\bar{a}}_{1}^{2}\left(s\right)]. \end{array}$$

To compute the effective coefficients, we truncate the system of Equation (54) to different orders of m=l and find $a_{1}^{d}\left(s\right)$ in (62a) and (62b). We notice that, to obtain the effective coefficients as functions of time, we need to apply the inverse Laplace-Carson transform. For this purpose, we adopt part of the scheme used in, e.g., [12] which employs the MATLAB function INVLAP [40].

### 8.1. Fibre Reinforced Composite with Elastic Inclusion and Kelvin-Voight Matrix

As a first specialisation of the developed theory, we consider the case of a composite with viscoelastic matrix of Kelvin-Voigt type and elastic inclusion. Specifically, for the matrix constituent, we assume that the memory function $\mu_{1}^{v}$ is given by the expression

(63) $$\begin{array}{c} \mu_{1}^{v}=\nu_{1}\delta \left(t\right), \end{array}$$

where $\nu_{1}$ represents the dynamic viscosity and δ(t) denotes Dirac’s delta. In this case, the effective coefficients ${\left[{\hat{G}}_{\mathrm{eff}}\left(s\right)\right]}_{3131}$ and ${\left[{\hat{G}}_{\mathrm{eff}}\left(s\right)\right]}_{3232}$, which are given in (62a) and (62b), take the form

(64a) $$\begin{array}{cc} \; & {\left[{\hat{G}}_{\mathrm{eff}}\left(s\right)\right]}_{3131}=[\mu_{1}^{e}+s\nu_{1}][1-2\pi {\bar{a}}_{1}^{1}\left(s\right)], \end{array}$$

(64b) $$\begin{array}{cc} \; & {\left[{\hat{G}}_{\mathrm{eff}}\left(s\right)\right]}_{3232}=[\mu_{1}^{e}+s\nu_{1}][1+2\pi {\bar{a}}_{1}^{2}\left(s\right)]. \end{array}$$

**Figure 3:**
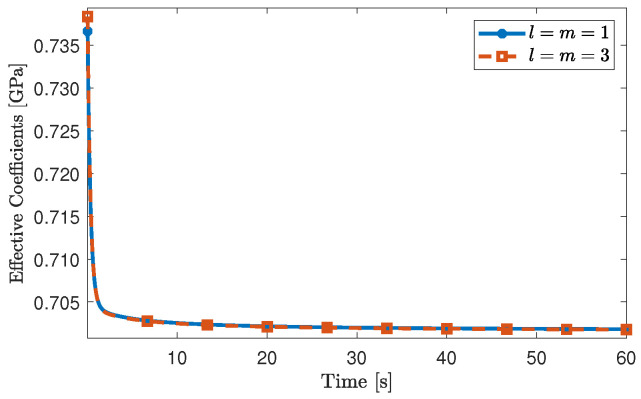
Effective coefficient ${\left[G_{\mathrm{eff}}\right]}_{3131}$ for two different orders of approximation. The volumetric fraction of the fibre inclusion, $V_{2}$, has been set equal to 0.1.

In Figure 3, we report the numerical values of the effective coefficient ${\left[G_{\mathrm{eff}}\right]}_{3131}$ with respect to time (that is, after computing the inverse Laplace–Carson transform) for different orders of truncation of the linear system. In particular, we choose the set of parameters $\mu_{1}^{e}=0.75$ GPa, $\mu_{2}^{e}=0.375$ GPa and $\nu_{1}=0.375$ GPa·s [41]. In particular, in Figure 3, we notice that for the truncation orders l=m=1 and l=m=3, the effective coefficients are almost indistinguishable, which suggests that the numerical computations converge rapidly, making them cost-efficient. Because of the good agreement shown in Figure 3, we continue our analysis with the truncation order l=m=1, unless indicated otherwise.

**Figure 4:**
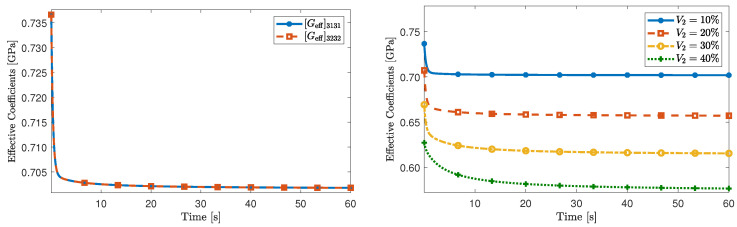
(**Left** panel) Effective coefficients ${\left[G_{\mathrm{eff}}\right]}_{3131}$ and ${\left[G_{\mathrm{eff}}\right]}_{3232}$ for $V_{2}=0.1$. (**Right** panel) Effective coefficient ${\left[G_{\mathrm{eff}}\right]}_{3131}$ for different values of the fibre’s volumetric fraction.

In Figure 4 (panel on the left), we show a comparison between the effective coefficients ${\left[G_{\mathrm{eff}}\right]}_{3131}$ and ${\left[G_{\mathrm{eff}}\right]}_{3232}$. As expected, from the material symmetry assumptions and the geometric setting, ${\left[G_{\mathrm{eff}}\right]}_{3131}$ is equal to ${\left[G_{\mathrm{eff}}\right]}_{3232}$. Furthermore, in Figure 4 (panel on the right), we report the time evolution of ${\left[G_{\mathrm{eff}}\right]}_{3131}$ for different values of the fibre volumetric fraction. Particularly, for the specific values considered in this work, for higher values of the fibre radius, the magnitude of ${\left[G_{\mathrm{eff}}\right]}_{3131}$ decreases. We also advert that as the fibre volumetric fraction decreases, the effective coefficient ${\left[G_{\mathrm{eff}}\right]}_{3131}$ tends to the shear modulus of the viscoelastic matrix, that is $\mu_{1}^{e}=0.75$ GPa. This behaviour agrees with the fact that, physically, the material becomes a viscoelastic homogeneous medium.

### 8.2. Fibre Reinforced Composite Characterised by Prony Series

In this second benchmark problem, we consider that both constituents are characterised by a Prony series. That is, we set

(65) $$\begin{array}{c} \mu_{\eta }^{v}\left(t\right)=\sum_{i=1}^{N}\mu_{\eta i}^{e}\exp\left(-t/\tau_{\eta i}\right), \end{array}$$

where $\mu_{\eta i}^{e}$ denote, for each i=1,2,…,N, the shear elastic modulus and $\tau_{\eta i}$ are the corresponding relaxation times. Consequently, the effective coefficients given in (62a) and (62b) can be written in the form

(66a) $$\begin{array}{cc} \; & {\left[{\hat{G}}_{\mathrm{eff}}\left(s\right)\right]}_{3131}=[\mu_{1}^{e}+\sum_{i=1}^{N}\frac{\mu_{1i}s}{s+\tau_{1i}^{-1}}][1-2\pi {\bar{a}}_{1}^{1}\left(s\right)], \end{array}$$

(66b) $$\begin{array}{cc} \; & {\left[{\hat{G}}_{\mathrm{eff}}\left(s\right)\right]}_{3232}=[\mu_{1}^{e}+\sum_{i=1}^{N}\frac{\mu_{1i}s}{s+\tau_{1i}^{-1}}][1+2\pi {\bar{a}}_{1}^{2}\left(s\right)]. \end{array}$$

To perform the numerical simulations, we consider the same parameters specified in [11], which for convenience are reported in Table 1. Specifically, the matrix is chosen as a soft viscoelastic material and the fibre as a stiff viscoelastic material.

**Figure 5:**
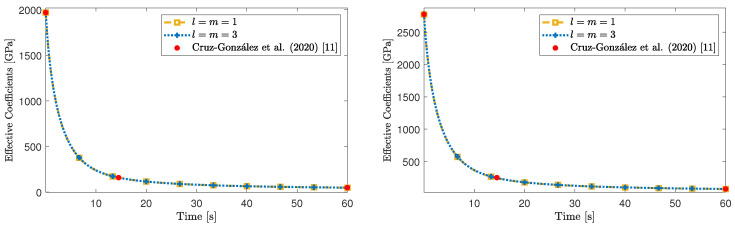
Comparison of the effective coefficient ${\left[G_{\mathrm{eff}}\right]}_{3131}$ with the numerical results reported in [11] for two different fibre volumetric fractions $V_{2}=0.1$ (panel on the **left**) and $V_{2}=0.3$ (panel on the **right**).

The results of our simulations are shown in Figure 5 and we compare them with those obtained in [11], in which the Authors used numerical simulations based on finite elements. Specifically, we select the numerical results reported in [11] for $V_{2}=0.1$ and $V_{2}=0.3$. These are represented as red dots in Figure 5 and agree with the values computed in this work.

**Figure 6:**
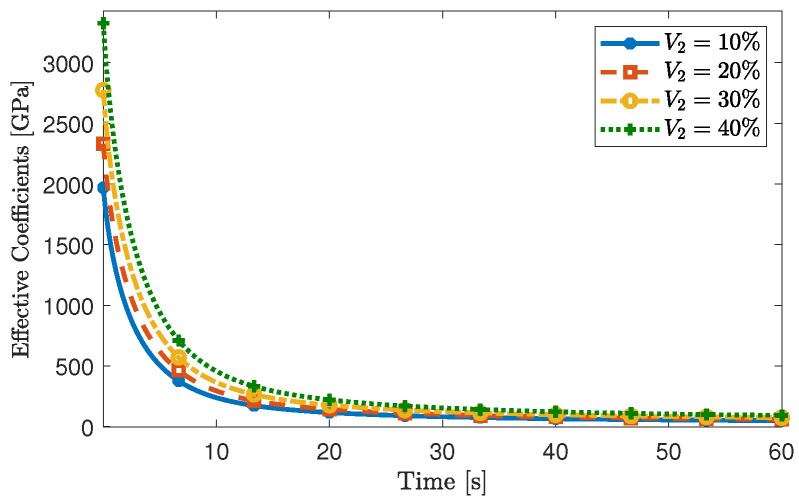
Influence of the fibre’s volumetric fraction on the effective coefficient ${\left[G_{\mathrm{eff}}\right]}_{3131}$.

Additionally, in Figure 6, we also report the time-dependent behaviour of the effective coefficient ${\left[G_{\mathrm{eff}}\right]}_{3131}$ for different values of the fibre volumetric fraction. While the differences in variations of the fibre diameter may not be as significant as in the previous benchmark problem, we still observe the influence of $V_{2}$. Predictably, the loss of the stiffness provided by the fibre produces a softening of the composite.

## 9. Conclusions

In this work, we proposed a general formulation for the study of nonlinear viscoelastic composites using the asymptotic homogenisation technique in a purely mechanical framework involving the equilibrium equation in the absence of body forces. After obtaining the local problems associated with the original formulation, we showed the potential of the scheme by considering the Saint–Venant strain energy density and formulating the cell and homogenised problems in the limit of infinitesimal deformations. This choice was mainly due to the numerical complications arising in a finite theory and the convenience of the use of the correspondence principle in linear problems. Furthermore, we analytically solved the anti-plane local problems associated with uniaxially fibre-reinforced materials and our results showed good agreement with published data. We remark that the model developed here is versatile and that, although only the effective coefficients for Kelvin–Voigt and Prony series models were reported, it can be easily adapted to different selections of viscoelastic laws.

As we mentioned before, the selection of the Saint–Venant strain energy density was mostly driven by its simplicity. Nevertheless, the methodology presented here could be adapted to other types of strain energy densities. However, this will limit the use of the correspondence principle and, consequently, appropriate numerical schemes need to be considered for the time integral (see, e.g., [42]). We further remark that the method to find the solution to the cell problems can be extended, for example, as done in [15], to other topological settings.

Further advancements in this work encompass a wider analysis of diverse types of nonlinear viscoelastic materials and contextualising the general theory in relevant biological scenarios. For instance, in the case of biological fibrous tissues, such as muscles and connective tissues. Further research in this area is expected to lead to new research questions in materials science and biomathematics.

## Figures and Tables

**Table 1:** Parameters used in the numerical simulations [11].

η=1			η=2		
$\mu_{1}^{e}$ [GPa]	$\mu_{1i}$ [GPa]	$\tau_{1i}$ [s]	$\mu_{2}^{e}$ [GPa]	$\mu_{2i}$ [GPa]	$\tau_{2i}$ [s]
3.162	2.512	0.032	100	3.162	3
	10	0.1		17.1783	10
	56.234	0.316		100	32
	316.228	1		316.228	100
	1000	3.162		1000	316
	199.526	10		5623.413	1000
	50.119	31.623		10,000	3162
	19.953	100		562.341	10,000
	12.589	316.228		141.254	31,623
	2.512	1000		56.234	100,000
	1.698	3162.278		17.783	316,228
	1.202	10,000		5.623	1,000,000
	1.148	31,622.777		3.162	3,162,278
	1.096	100,000		1.778	10,000,000
